# A Magnetorheological Duckbill Valve Micropump for Drug Delivery Applications

**DOI:** 10.3390/mi13050723

**Published:** 2022-04-30

**Authors:** Rubayet Hassan, Sevki Cesmeci, Mahmoud Baniasadi, Anthony Palacio, Austin Robbins

**Affiliations:** 1 Department of Mechanical Engineering, Georgia Southern University, Statesboro, GA 30460, USA; rh17100@georgiasouthern.edu (R.H.); ar08678@georgiasouthern.edu (A.R.); 2 Intel Corporation, Hillsboro, OR 97124, USA; mahmoud.baniasadi@intel.com; 3 Department of Manufacturing Engineering, Georgia Southern University, Statesboro, GA 30460, USA; ap07917@georgiasouthern.edu

**Keywords:** micropump, magnetorheological, MRE

## Abstract

In this study, we propose a duckbill valve microfluidic pump that relies on an electromagnetic actuation mechanism. An FEA/CFD-based approach was adopted for the design of the device due to the coupled electromagnetic–solid–fluid interactions in the device. The simulation methodology was confirmed with the previously published data in the literature to ensure the accuracy of the simulations. The proposed optimum duckbill valve micropump can pump 2.45 µL of fluid during the first 1 s, including both contraction and expansion phases, almost 16.67% more than the basic model. In addition, the model can pump a maximum volume of 0.26 µL of fluid at the end of the contraction phase (at 0.5 s) when the magnetic flux density is at maximum (0.027 T). The use of a duckbill valve in the model also reduces the backflow by almost 7.5 times more than the model without any valve. The proposed device could potentially be used in a broad range of applications, such as an insulin dosing system for Type 1 diabetic patients, artificial organs to transport blood, organ-on-chip applications, and so on.

## 1. Introduction

There is an increasing demand in microfluidic flow control devices in a wide range of applications, including point-of-care [1] and lab-on-a-chip industries [2]. Although they are compact in size, microfluidic chips are equipped with bulky supply systems that are connected with microbore tubing to provide air pressure or reagent [3,4,5,6,7]. Although such systems provide precision pumping capabilities, the increased complexity hinders their applications in specific industries such as the welfare industry, in which a portable, wearable, and patchable insulin delivery system could be utilized to enhance the well-being of type 1 diabetic patients.

There have been various micropump designs studied in the literature. The piezoelectric-, magnetic-, electrochemical-, acoustic-, and electrothermal-based micropump have their positive and negative sides over one another in terms of scalability, biocompatibility, complexity, and accuracy, cost, and reliability [8]. For instance, lead zirconate titanate (PZT)-based micropumps offer a precise volumetric flow rate of aqueous fluids at fast response times and high actuation forces. However, they are complex to manufacture, require special care when installing their intricate components, such as the PZT discs, and demand high actuation voltages to operate. However, the electrochemical-based micropumps are relatively simpler to fabricate, offer continuous and smooth fluid delivery, can provide relatively larger displacements at lower power consumption, and can have battery-less versions when equipped with triboelectric nanogenerators. As for their drawbacks, they generate bubbles, which can subsequently dissolve into the working fluid, causing instabilities during pumping. Apart from these, various magnetic-based micropumps have been studied in the literature [9,10,11,12]. Some operate on the principle that an external magnetic field displaces a membrane mechanically to push the fluid forward. In contrast, others, such as magnetohydrodynamic (MHD) micropumps, rely on the Lorentz force under the combined effect of the electric and orthogonal magnetic fields to create the pumping effect.

Magnetic-based designs that rely on mechanical displacement of a membrane can be categorized into two groups: (i) designs wherein the membranes are pushed by a set of actuator heads to create a pressure differential, and (ii) designs that deform a ferromagnetic composite material, usually consisting of a polydimethylsiloxane (PDMS)-based matrix and micro- or nano-sized ferromagnetic iron particles embedded in them, under an externally applied magnetic field [8]. Designs from the first category are complex and have excess moving components, which could become a concern for installation and maintenance. The second category of pumps is more design-dependent. Although some designs feature a ferromagnetic membrane that deflects into a disk-shaped reservoir to generate the pumping effect, others contract a circular or rectangular flow channel for a pulsated flow. Using a magnetic field as a trigger has several advantages over other modes of operation. Magnetic actuators can generate strong force and displacement with lower power consumption, and they are less prone to electrical heat loss and high-voltage failure. Moreover, the actuation parts and magnetic field-generating parts can be physically separated, so these systems do not require complex wiring. Magnetorheological elastomers (MREs) are composite materials consisting of a rubber-like base material and micron-sized iron particles doped in it [13]. They can be designed as isotropic and non-isotropic, meaning that the magnetic iron particles inside them can be aligned in special configurations so the material responds to the applied loads differently. As such, these materials have found very wide application areas, ranging from vibration mounts in automobile engines to bearings in large building structures and bridges as well as sensor applications for structural health monitoring purposes, among other areas. Their application in the micropump field has not been overlooked. Various MRE micropump designs have been proposed in the literature [14,15,16,17,18,19,20,21,22,23]. Although some featured one-way valves for unidirectional pumping effect [22], others used a series of electromagnets to transport the fluid through the pump channel [24,25,26]. Behrooz and Gordaninejad applied a soft MRE membrane for conveying Newtonian fluid to understand the microfluid transportation system, and they also demonstrated the performance is significantly affected by the design parameters [27,28]. Stork was the first to investigate the influence of electromagnets on fluid transport and an MRE peristaltic pump to convey fluid [29]. Ehsani and Nejat proposed a simple conceptual design of a flexible-valve micropump based on using magneto-fluid-structural interaction (MFSI) three physics simulations [30]. Xufeng et al. proposed using an MRE-based magneto-active pulse pump in 3D, but they did the numerical analysis without using valves inside the microchannel [31]. These existing micropump designs have their own limitations and/or drawbacks. For instance, the initial concept proposed by Behrooz and Gordaninejad provides lower pumping capacity. The micropump design proposed by Ehsani and Nejat operates with a relatively slower response time under the magnetic field and also provides a weaker actuation force. Moreover, their design suffers from large backflow issues due to the larger gap between the tip of the valves and the upper wall. The design proposed by Xufeng et al. experiences slower response time and lower pumping capacity as well. Therefore, there is still room for novel pump magnetorheological pump designs that could potentially operate with faster response times, minimize the backflow, and provide higher pumping capacities.

It is a common agreement that fabrication of such designs prove to be challenging at microscale. However, with the recent advancements in 3D-printing technology, manufacturing possibilities of such designs are revisited. As such, fabrication aspects should be taken into consideration during the design stage.

In the light of above discussions,

- we propose a novel magnetorheological peristaltic micropump that has not been studied previously to offer an efficient, miniature (on the order of 1 mm), lightweight, portable, wirelessly controllable (with a fast response time of less than 100 ms), durable, low-power micropump for drug delivery; and we believe that
- originality of the study stems from the fact that the proposed design is not only novel but is also designed to be fabricated by 3D-printing technologies in a more convenient way than the existing MRE micropumps in the literature.

The details of the design will be discussed in Section 2. A multiphysics-based simulation approach was adopted to prove the proposed concept. Highly coupled magneto-solid-fluid interaction simulations were carried out in COMSOL Multiphysics software (v5.6). To investigate the effects of significant design parameters, a parametric analysis was also conducted. The paper is organized as follows: the proposed design is discussed in Section 2, simulation methodology is presented in Section 3, parametric and optimization studies are covered in Section 4, and summary and conclusions are discussed in Section 5.

## 2. Proposed Design

**Figure 1:**
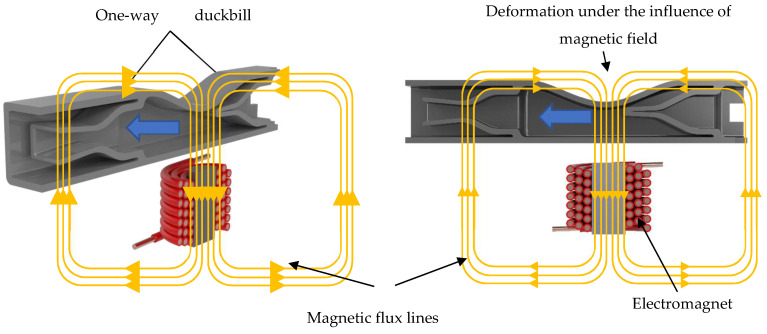
The proposed micropump design with its main components.

The proposed design is illustrated in Figure 1. It consists of a pumping chamber, two one-way duckbill valves, and an electromagnet. The top wall of the pumping chamber is made from a semi-active material called magnetorheological elastomer (MRE), whereas the rest of the structure, including the valves, consists of a passive elastomer. MREs are categorized as composite materials as they are composed of an elastomeric (or polymeric) matrix, such as silicon, with micron-sized, magnetically permeable particles, typically iron particles, doped in it. Due to their ferromagnetic properties, MREs deform under an external magnetic field. In contrast, they can also be designed to resist the deformation by activating an external magnetic field. This unique feature of MREs allowed them to be utilized in a wide range of application areas including, actuation systems, vibration isolation systems, and sensors. In the proposed design, the top wall contracts toward the pumping chamber under the magnetic field. The magnetic field and thus the amount of contraction is controlled by an electromagnet placed underneath the pump. The contraction of the top wall increases the pressure by constricting the fluid inside the flow chamber, forcing the fluid through the one-way valve in the front end. Simultaneously, the other duckbill valve in the rear experiences backpressure, which forces the geometry to seal itself, preventing the fluid from leaking backward. This one-way flow creates an effective pumping mechanism as it continually sends the fluid forward.

## 3. Simulation Methodology

### 3.1. Model Creation

**Figure 2:**
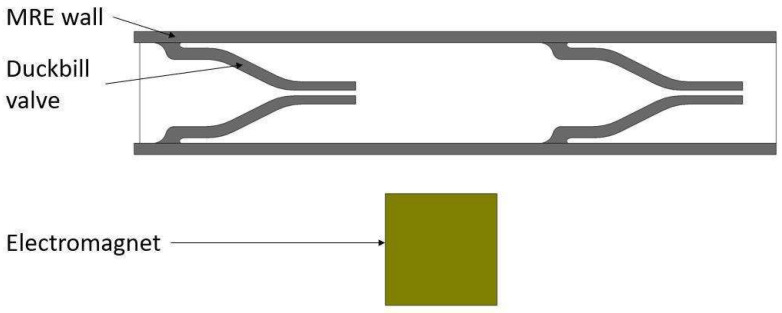
Duckbill valve model with its main components.

The proposed duckbill valve design involves coupled magneto–solid–fluid interaction physics. Thus, the performance of the pump could best be predicted with the help of computer simulations rather than simplified 1D analytical models. In this study, simulations were carried out by using COMSOL Multiphysics software. Figure 2 shows a schematic of the model created in COMSOL. To demonstrate the effectiveness of the flap valve, simulations were conducted with and without valves. 

**Figure 3:**
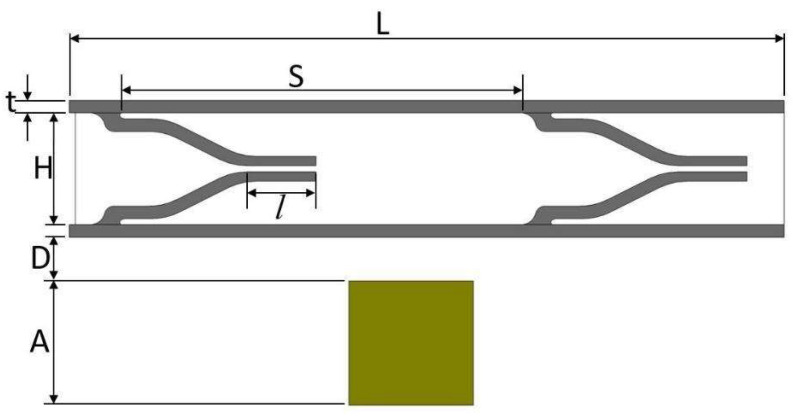
Duckbill valve model in 2D with its main dimensions.

The geometric and material properties of the 2D model are given in Table 1, and the dimensions are shown in Figure 3 for convenience. The magnet is placed 0.35 mm below of the pump chamber. The length, height, and wall thickness of the pump chamber are 3.102 mm, 1.100 mm, and 0.100 mm, respectively. The elastic modulus of the pump material and the average magnetic flux density acting on the upper wall are 1.2 MPa and 0.027 T, respectively.

**Figure 4:**
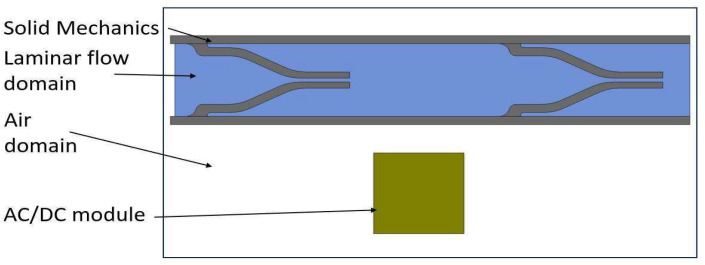
2D simulation model geometry.

In this study, the MRE is assumed to be a ferromagnetic material that has constant structural and magnetic properties. The wall at the bottom does not deform in the presence of a magnetic field. It is assumed to be resting on a flat rigid surface so the deformation on the top wall can activate the fluid flow. The magnetic field analysis was conducted in an AC/DC module, whereas the structural deformation of the pump chamber, including the top wall and one-way flap valves, and fluid flow through the pump chamber and valves were carried out in solid mechanics and laminar flow modules, respectively (Figure 4). 

### 3.2. Simulation Procedure

**Figure 5:**
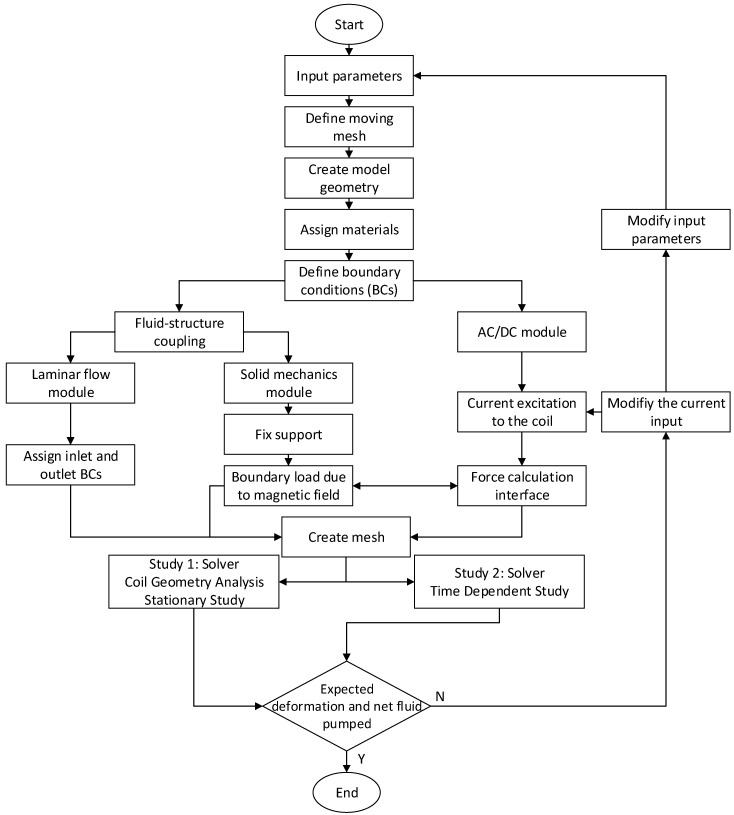
Flowchart of the simulation process in COMSOL.

The flowchart of the simulation procedure is shown in Figure 5. First, the parameters were defined, such as geometric and material properties. Next, the moving mesh schemes had to be defined because the simulation involved the deformation of the pump chamber and one-way valves. Then the geometry used for the simulation was created, followed by the material assignment to all solid and fluid domains. After that, respective boundary conditions were assigned in each physics module (AC/DC, solid mechanics module, and laminar flow). The different physics modules then transferred data between the different flow physics. For example, the upper wall of the pump chamber undergoes a downward deflection under the influence of a magnetic field because it consists of a ferromagnetic material. To model this phenomenon, the AC/DC module was run to calculate the magnetic field over the entire domain, including all solid and fluid domains. The information was then passed over to the solid mechanics module to calculate the deformation under the magnetic field. Likewise, the deformation data from the top wall was transferred to the fluid domain via fluid-structure coupling between the solid mechanics and laminar flow modules. The fluid-structure coupling was a two-way coupling that provided communication back and forth between the fluid and solid domains. This communication was accomplished by defining fluid-structure interaction multiphysics at all interfaces between the fluid and solid domains. The deformation due to the magnetic field presented itself as a pressure load in the fluid domain at the fluid–solid interface, creating the pumping effect. Although the fluid was pushed through the front valve, the pressure data from the fluid domain was sent back to the solid structures through the fluid-structure coupling, and vice versa.

### 3.3. Boundary Conditions

When applying the boundary conditions, the AC/DC module was used to apply a current excitation to the coil, and the force calculation interface was selected for the top edge of the upper wall of the pump chamber. This placement at the top edge ensured that the Maxwell forces were transferred to the top edge for the desired deformation and thus, the desired pumping effect. The Maxwell surface stress tensor (the magnetic interaction force within the pump chamber caused by the magnetic field) was selected in the boundary load interface, i.e., the top edge of the top wall. An external current density, with a specified amplitude, as well as a sinusoidal time function were applied to the magnet core. A fixed support was placed on the bottom wall inside the pump chamber by using the solid mechanics module. As for the laminar flow module, the relative pressures at outlets of the pumping system were set to 0 Pa (gage). This allows the fluid to freely pass through the inlet and outlet sections. Finally, a no-slip condition was assigned to the walls surrounding the flow chamber.

### 3.4. Computing Equations

Next, the governing physics equation is discussed. The following equations were solved for the magnetic domain:

(1) ∇·J=0

(2) ∇×H=J

(3) $$B=\mu_{0}\mu_{r}H$$

(4) $$J=\nabla \times {\left(\mu_{r}\mu_{0}\right)}^{-1}B,$$

where  J is the current density, ∇ is the gradient operator, B is the magnetic flux density; $\mu_{0}$ and $\mu_{r}$ are the permeability of the vacuum and the relative magnetic permeability. Equation (1) used Ampere’s Law to determine the magnetic flux density. Through the combination of Equations (1)–(3), the relation between J and B can be obtained, which is shown by Equation (4),

(5) $$J=\sigma E+\sigma v\times B+J_{e}$$

where E is the electric field, σ is the electrical conductivity, v is the velocity of the conductor, and $\;J_{e}$ is the external current density. The magnetic flux density B and external current density $J_{e}$ can be calculated by using Equations (6) and (7).

(6) B=∇×A

(7) $$J_{e}=\frac{I\cdot n}{a\cdot b}$$

In Equations (6) and (7), A is the magnetic vector potential, I is the input electrical current to the coil, n is the number of turns of the coil of the electromagnet, and variables *a* and *b* are the cross-sectional dimensions of the magnetic core. Once the external current density $J_{e}$ is known, the magnetic field density B can be calculated by using Equation (8).

(8) $$J_{e}=\nabla \times {\left(\mu_{r}\mu_{0}\right)}^{-1}B-\sigma E-\sigma v\times B$$

To calculate the Maxwell stress, Equation (9) can be used. It can then be inserted into Equation (10) where it is added with the stress due to fluid pressure to find the total stress.

(9) $$n.\sigma_{maxwell}=-0.5n\left(H\cdot B\right)+\left(n\cdot H\right)B^{T}$$

(10) $$\sigma_{total}=\sigma_{maxwell}+\sigma_{p},$$

where n is the unit normal vector, $\sigma_{total}$ is the total stress, $\sigma_{Maxwell}$ is the Maxwell stress, and $\sigma_{p}$ is the stress due to fluid pressure. When considering the fluid domain, laminar incompressible flow was set as the flow type. The fluid domain was then solved by using the following equations:

Steady continuity:

(11) $$\rho \nabla \cdot u_{fluid}=0$$

Navier-Stokes equation used in the stationary approach:

(12) $$\rho \left(u_{fluid}\cdot \nabla \right)u_{fluid}=\nabla \cdot \left[-pI+K\right]+F$$

Navier-Stokes equation used for the time-dependent approach:

(13) $$\rho \frac{\partial u_{fluid}}{\partial t}+\rho \left(u_{fluid}\cdot \nabla \right)u_{fluid}=\nabla \cdot \left[-pI+K\right]+F,$$

where ρ is the density, $u_{fluid}$ is the velocity vector, p is the pressure, K is the turbulent kinetic energy, and F is the volume force vector. Equation (14) can be used to find the turbulent kinetic energy K.

(14) $$K=\mu (\nabla u_{fluid}+{\left(\nabla u_{fluid}\right)}^{T})$$

Then finally for the solid mechanics domain, the following equations were solved:

Equation for the stationary approach:

(15) $$0=\nabla \cdot {\left(FS\right)}^{T}+Fv,\;F=l+\nabla u_{solid}.$$

Equation for the time-dependent approach:

(16) $$\rho \frac{\partial^{2}u_{solid}}{\partial t^{2}}=\nabla \cdot {\left(FS\right)}^{T}+Fv,\;F=l+\nabla u_{solid}.$$

In the above equations, Equations (15) and (16), *FS* (*F* is the deformation gradient) is the first Piola–Kirchhoff stress tensor, Fv is the volume force vector, *l* is the unit tensor, and $u_{solid}$ is the displacement.

### 3.5. Grid Generation and Mesh Independence Study

**Figure 6:**
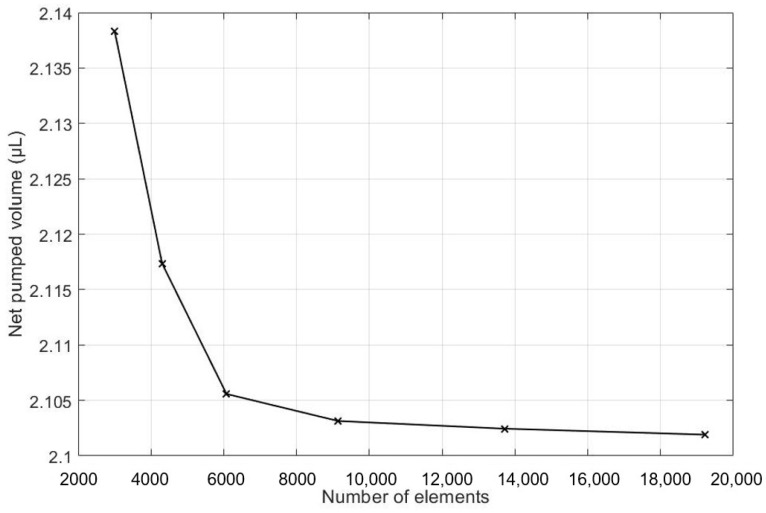
Net volume pumped is calculated for different numbers of elements.

As is commonplace in procedures for conducting simulations in both finite element analysis (FEA) and computational fluid dynamics (CFD), a mesh independency analysis was conducted. To begin this process, a course mesh was applied to an initial simulation. Then, the mesh size was reduced continually, and the net pumped volume was monitored after each run. The mesh size reduction continued until there was no significant change between two sequential cases. This process is shown in Table 2, which is also visually illustrated in Figure 6. From the recorded cases, it can be seen that the percent change in the target parameter was 0.14% between grid numbers 4 and 5. Thus, grid number 4 is selected for the rest of the simulations. 

### 3.6. Validity Study

**Figure 7:**
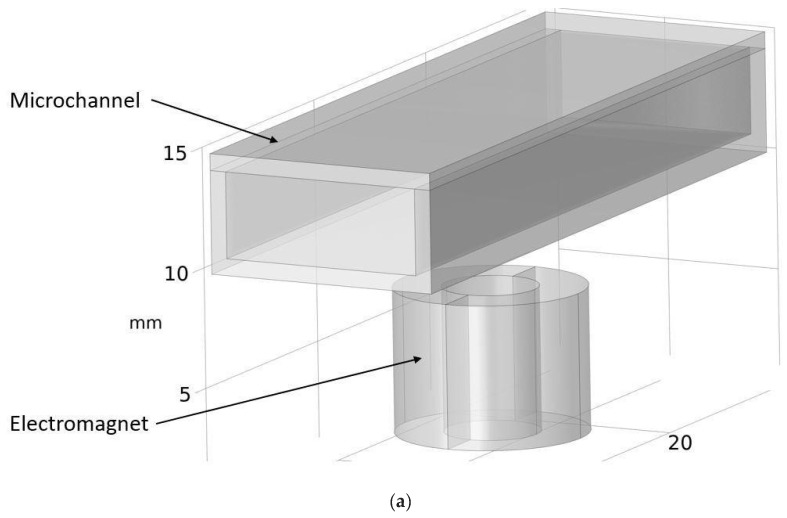

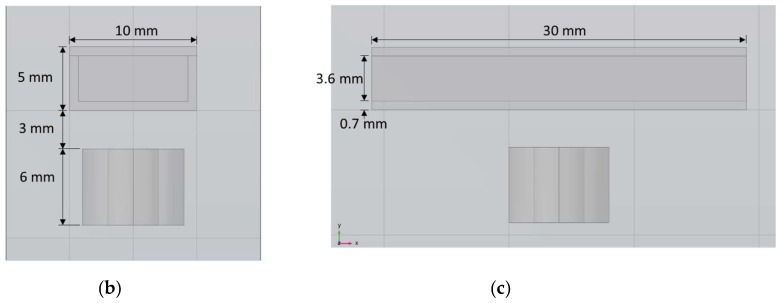
Schematic of the model studied in [31]: (**a**) 3D view. (**b**) Front view. (**c**) Side view.

Although COMSOL Multiphysics is a proven simulation tool and has been employed by thousands of scientific studies in the literature, it is always wise and scientifically required to validate the simulation approaches with the existing studies in literature when experimental data is not readily available. To this end, we selected the model presented in [31]. All parameters, boundary conditions, magnetic flux density, and geometric dimensions were set to be the same. Figure 7 shows the model with the main components as well as the dimensions.

**Figure 8:**
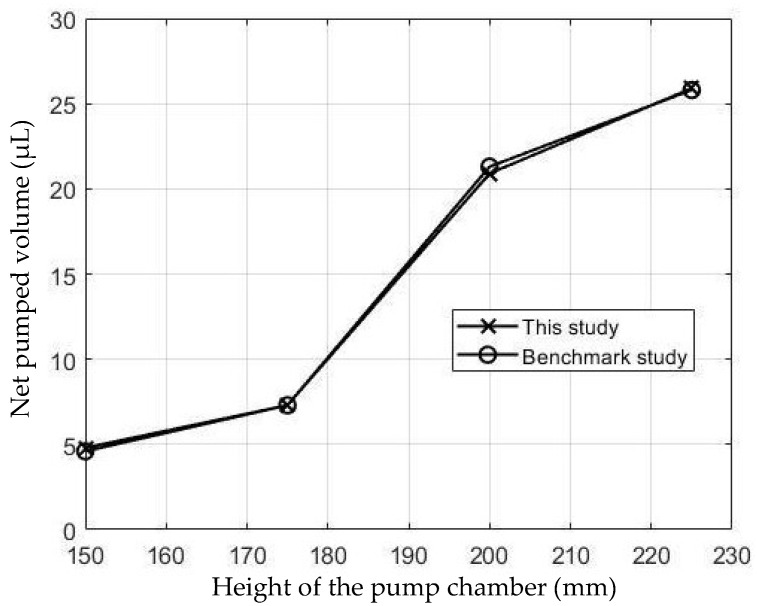
Net pumped volume comparisons between this study and benchmark study [31].

The simulations were carried out by following the procedure outlined in Figure 5. The comparisons between the benchmark study and this study are given in Table 3. From Table 3, it is seen that the upper wall displacement for both cases is the same for 75 mT, 145 mT, and 175 mT magnetic flux densities. In addition to the deformations, volume flow rates were also compared. Figure 8 presents a comparison graph between the two cases. As seen from the figure, the volume flow rates closely matched between the two cases. Further, the numerical data for the volume flow rates are presented in Table 4 with percent error margins. It can be seen that the average percent error between the two cases is about 1.6%, with minimum and maximum deviations being 0.00% and 4.16% at 150 mT and 175 mT, respectively. This validates our simulation methodology, allowing us to continue with the full simulations of the proposed pump design. 

### 3.7. Results and Discussions

**Figure 9:**
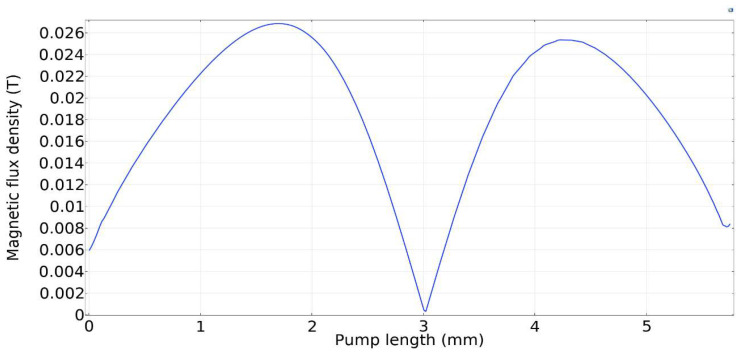
Magnetic flux density on the upper MRE wall and across the pump length.

Simulations were performed for a complete cycle, i.e., for a full contraction and retraction phases. Based on the literature, we used a magnetic field input of 0.027 T. The magnetic field acts on the magnetically permeable particles within the MRE, creating a downward force on the upper wall. The magnetic flux density on the upper wall along the length of the microchannel is given in Figure 9. The figure shows that the magnetic flux density reaches its peak at the two terminals of the microchannel and its minimum value in the middle. This agrees with our intuitions because the magnetic field concentrates on the upper MRE wall before returning to the electromagnet to complete the magnetic circuit. Similar phenomena were also observed and reported in the literature [27,28,30,31].

**Figure 10:**
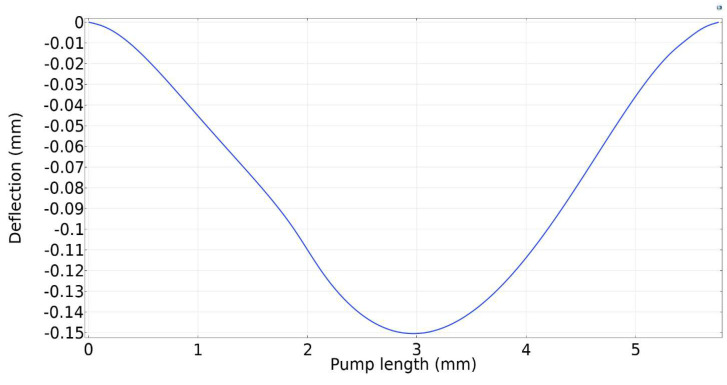
Deformation of upper wall across the pump length.

Figure 10 shows the deformation of the upper wall under the influence of the magnetic field. The maximum deflection was calculated to 0.11 mm, which occurred in the middle of the upper wall as expected.

**Figure 11:**
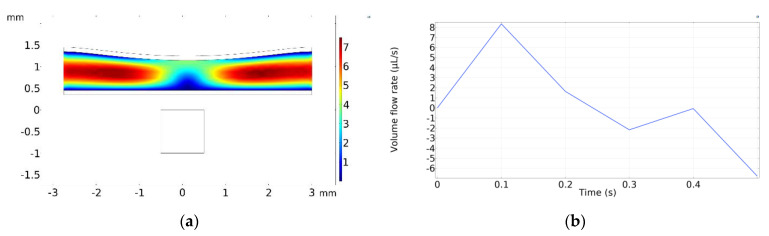
Simulation results for micropump without any valves. (**a**) Velocity field at *t* = 0.1 s. (**b**) Total volume flow rate vs. time.

Figure 11a displays the velocity field inside the pump at *t* = 0.1 s, when the maximum velocity occurs, whereas Figure 11b shows the total volume flow rate with respect to time. The total volume flow rate is calculated by summing the outflows from both the left and right sides. It should be noted that there is no inlet to the pump because the flow is initiated by the squeezing effect under the magnetic field.

**Figure 12:**
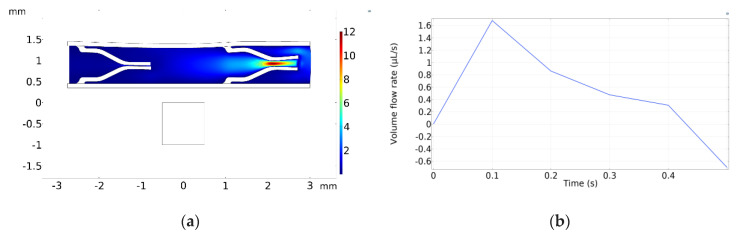
Simulation results for micropump with duckbill valves. (**a**) Velocity field at *t* = 0.1 s. (**b**) Volume flow rate vs. time.

Figure 12 displays the results for the same simulation configurations as Figure 11. However, in Figure 12, one-way duckbill valves were added inside the pump chamber. A quantitative comparison between Figure 11b and Figure 12b reveals that the inclusion of the duckbill valves reduced the backflow by about 7.5 times.

**Figure 13:**
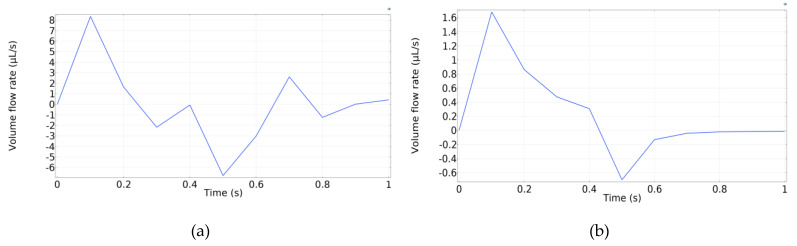
Volume flow rate. (**a**) Without valves. (**b**) With duckbill valves.

Figure 13 exhibits the volume flow rate during both contraction and retraction phases for (a) without valves and (b) with the duckbill valves. The figure shows that with the addition of duckbill valves, the net backward flow reduced significantly during the retraction phase with the addition of duckbill valves.

**Figure 14:**
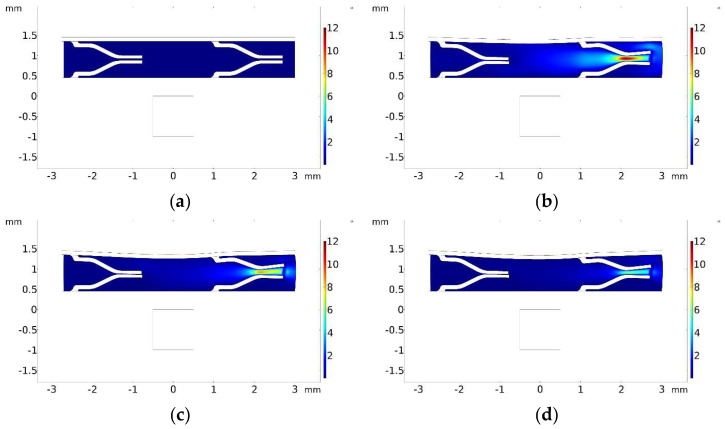

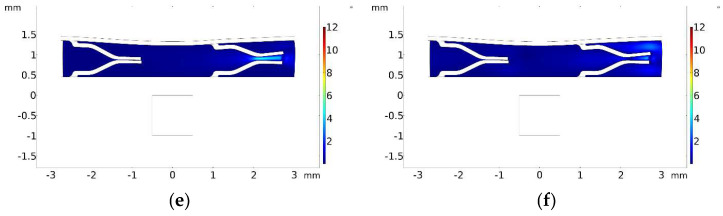
The deflection and velocity field history of the micropump during the contraction phase at *t* = 0–0.5 s. (**a**) The flow field at the beginning of the simulation at *t* = 0 s. (**b**) The MRE is started deforming downward, pushing the fluid toward the right terminal. The fluid shows maximum velocity at the right valve in this stage. (**c**) the upper wall deforms further, and thus, the right duckbill bends, but this time the tips of the right valve come closer to each other, thus decreasing fluid displacement (*t* = 0.2 s). (**d**) The right duckbill valve deflects further and reduces the fluid flow, and the left valve closes completely to prevent the backward flow (*t* = 0.3 s). (**e**) Deformation of the upper wall and duckbill valves continue (*t* = 0.4 s). (**f**) the deflections on the upper wall (*t* = 0.5 s). At this time, the micropump is about to start the expansion phase.

The displacement and velocity field history of the micropump is shown in Figure 14a–f for the contraction phase, i.e., *t* = 0–0.5 s. One can see that the right duckbill deforms and propels the fluid, whereas the left duckbill valve closes and blocks the backflow. Figure 14a shows the flow field at the beginning of the simulation at *t* = 0 s. In Figure 14b, the MRE is started deforming downward, pushing the fluid toward the right terminal. Due to a large amount of fluid being enclosed in between the two valves, the fluid shows maximum velocity at the right valve in this stage. In Figure 14c, the upper wall deforms further, and thus, the right duckbill bends, but this time the tips of the right valve come closer to each other, thus decreasing fluid displacement (*t* = 0.2 s). In Figure 14d, the right duckbill valve deflects further and reduces the fluid flow, and the left valve closes completely to prevent the backward flow (*t* = 0.3 s). In Figure 14e, deformation of the upper wall and duckbill valves continue (*t* = 0.4 s). In Figure 14f, the deflections on the upper wall (*t* = 0.5 s). At this time, the micropump is about to start the expansion phase.

**Figure 15:**
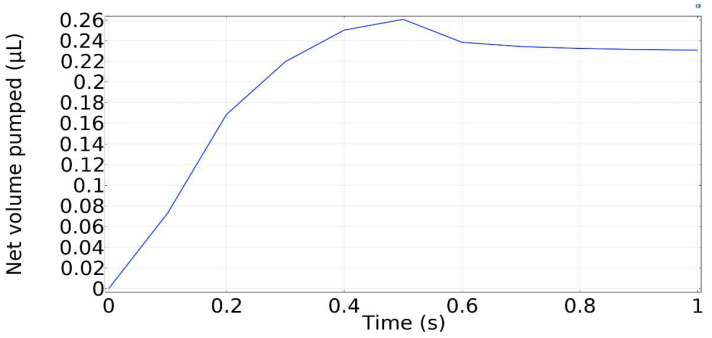
Net pumped volume.

The time history of net volume transferred through the right terminal of the micropump is displayed in Figure 15. During the contraction phase, the net volume transferred constantly increased until the contraction phase ends at *t* = 0.5 s. Following this, the channel begins retracting, and the net pumped volume decreases.

## 4. Parametric Study

The effects of important input parameters on the design target are also essential. Recalling that our primary goal was to increase the pumping effect of the proposed design, we carried out a parametric analysis to see the effects of various geometric and material properties, including the diameter of the pump chamber, the thickness of the upper MRE wall, the elastic modulus of the pump structure, and other geometric dimensions of the pump, on the net pumped volume. These input parameters can be found in Table 5 with their respective ranges.

### Parametric Simulation Results and Discussions

**Figure 16:**
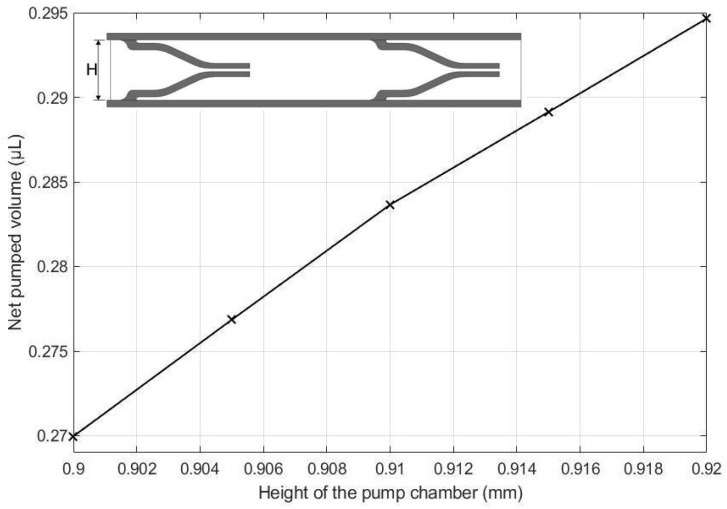
Effect of channel height on the net pumped volume.

**Figure 17:**
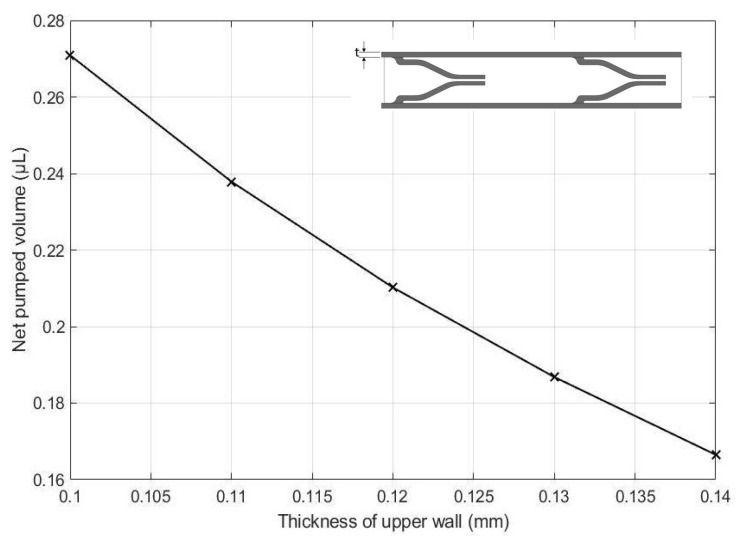
Effect of the thickness of the upper wall on the net pumped volume.

**Figure 18:**
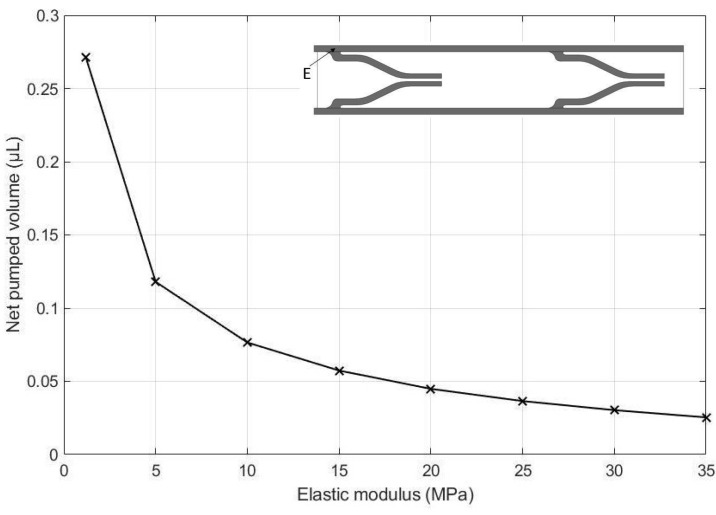
Effect of MRE elastic modulus on the net pumped volume.

**Figure 19:**
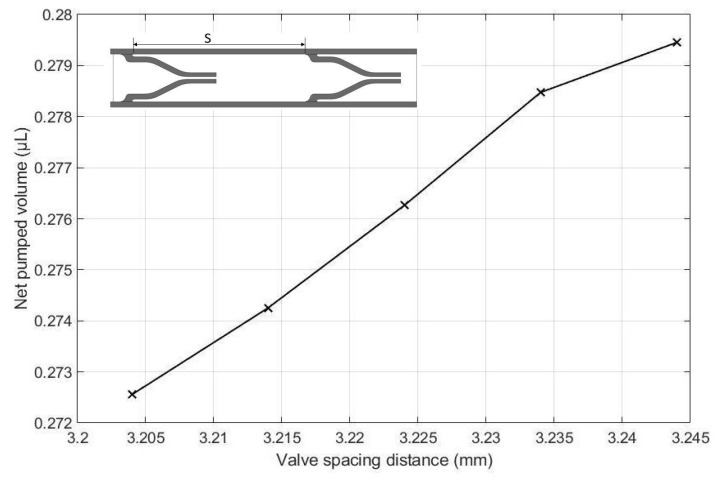
Effect of valve spacing distance on the net pumped volume.

**Figure 20:**
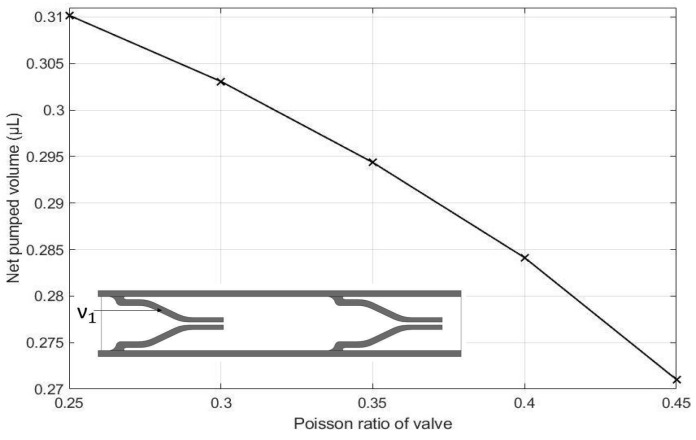
Effect of Poisson’s ratio of valve on net pumped volume.

**Figure 21:**
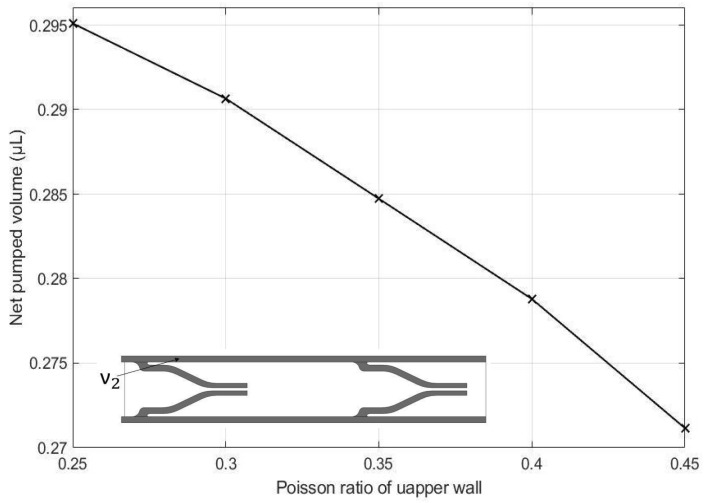
Effect of Poisson’s ratio of the upper wall on the net pumped volume.

**Figure 22:**
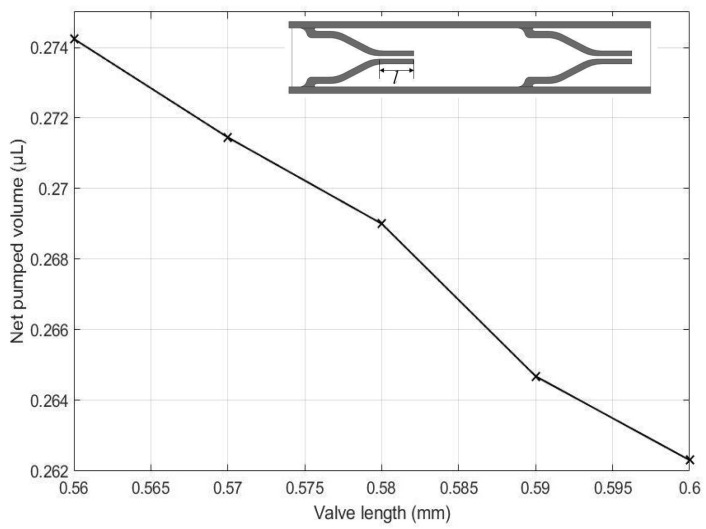
Effect of valve length on the net pumped volume.

The effects of the height of the pump chamber, thickness of the upper wall, elastic modulus of the pump structure, valve spacing distance, Poisson’s ratio of the valve, Poisson ratio of upper wall, and valve length on the net pumped volume are displayed in Figure 16, Figure 17, Figure 18, Figure 19, Figure 20, Figure 21 and Figure 22. The parametric sweep studies were performed in a way that the parameters left unchanged were set to their design values. These design values can be viewed in Table 5.

The net pumped volume increases with the height of the pump chamber which is exhibited in Figure 16. This increase is not only due to the pump chamber increase but also in part due to the modified aspect ratio of the flow chamber allowing larger deformation of the upper wall. This aligns with our intuitions.

In Figure 17 the variation of the net pump volume with respect to the thickness of the upper wall is shown. As displayed in the figure, the net pumped volume is decreasing with increasing values of the wall thickness. This makes sense as it becomes harder for the top wall to bend downward to create the pumping effect with larger wall thicknesses.

The effect of elastic modulus of the pump material on the net pumped volume is given in Figure 18. As seen in the figure, the net pumped volume decreases with increasing elastic modulus. This would agree with our intuitions because the larger the elastic modulus becomes, the stiffer the wall will be, resisting the deformation more strongly. 

The influence of valve spacing distance on the net pumped volume is shown in Figure 19. As seen in the figure, the net pumped volume of the fluid also increases with increasing valve spacing. This is because as the valve spacing becomes wider, a greater amount of fluid can be housed in between the two valves, resulting in more fluid volume being pumped, and thus enhancing the pumping effect. 

Figure 20 represents the effect of Poisson’s ratio on the valves. This also agrees with our intuitions because according to Hooke’s law, increased values of the Poisson’s ratio will result in decreased deformation of the valves, resulting in a lower pumping effect.

The influence of the upper wall’s Poisson’s ratio on the net pumped volume can be seen in Figure 21, which yields similar results to those shown in Figure 20. For this case, the increase in the Poisson’s ratio results in a decrease in the deformation experienced by the upper wall, deteriorating the pumping effect.

The effect of the valve length on the pumping performance in terms of the net pumped volume is shown in Figure 22. As seen, the net pumped volume continually decreases as the length of the valve increases. This is because the longer the valve is, the higher the frictional losses are, reducing the pumping effect. 

Parametric study revealed that each parameter contributed to the overall performance of the pump. Thus, an optimization study is necessary to determine the optimal values of these parameters for the maximum net pumped volume. To this end, we carried out an optimization study letting the material properties remain constant and changing only the geometric parameters. Table 6 shows the optimum values of the valve spacing distance and height of the pump chamber, which were the only two parameters that were changed from the original design values. The optimal design was then compared with the original design, the results of which are shown in Table 7. The comparisons revealed that the optimal design was capable of transferring 16.67% more fluid than the original model in one full cycle. 

## 5. Summary and Conclusions

In this paper, a comprehensive design methodology of a novel magnetorheological (MR) micropump was presented. To accomplish this, a physics-based simulation methodology was adopted. The validity of these simulations was checked by replicating an existing study in the literature. The dynamic performance of the pump was obtained and analyzed. Also, a parametric study was conducted to see the influence of each design parameters on the pump performance, which was then used for an optimization study. Major findings of this study can be listed as follows.

- A novel micropump design was successfully demonstrated.
- a parametric study revealed that the net pumped volume increased by 0.66% as the height of the pump chamber increased from 0.90 mm to 0.92 mm, decreased by 2.83% as the thickness of the upper wall increased from 0.10 mm to 0.14 mm,
- decreased by 6.66% as the elastic modulus of the pump structure increased from 1.2 MPa to 35 MPa,
- increased by 0.19% as the valve spacing distance increased from 3.204 mm to 3.244 mm,
- decreased by 1.21% as the Poisson ratio of valve increased from 0.25 to 0.45,
- decreased by 0.7% as the Poisson ratio of upper increased from 0.25 to 0.45, and
- decreased by 0.33% as the valve length increased from 0.56 mm to 0.60 mm.
- As a result of an optimization study, an optimum design is proposed, which can transfer 16.67% more fluid than the original model.
- The proposed micropump technology can be utilized in a wide range of application areas, including drug delivery systems, artificial hearts, in situ cell sorting and cytometry in laboratory environments, and thermal management techniques on PCBs. The proposed design methodology serves as a foundation for future MR micropump designs.

## Figures and Tables

**Table 1:** Properties of duckbill valve model.

Parameter	Symbol	Value
Height of the pump chamber	*H*	0.900 (mm)
Thickness of the upper wall	*t*	0.100 (mm)
Valve spacing distance	*S*	3.204 (mm)
Length of micro channel	*L*	5.650 (mm)
Distance between the pump chamber and electromagnet	*D*	0.350 (mm)
Length of the valve tip	*l*	0.560 (mm)
Side of the electromagnet	*A*	1.000 (mm)
Magnetic flux density	*B*	0.027 (T)
Elastic modulus	*E*	1.200 (MPa)

**Table 2:** Net pumped volume for each grid number.

Grid Number	Number of Mesh Elements	Net Pumped Volume (µL)	The Difference in Net Pumped Volume (%)
1	3002	2.13832	-
2	4306	2.11735	0.98
3	6075	2.10558	0.56
4	9144	2.10314	0.51
5	13708	2.10245	0.14
6	19215	2.10191	0.11

**Table 3:** Comparisons of simulations between this study and benchmark study.

Magnetic Flux Density (mT)	Benchmark Study [31]	This Study	Maximum Displacement of Upper Wall (mm)
75	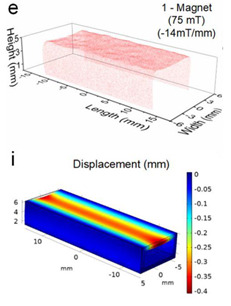	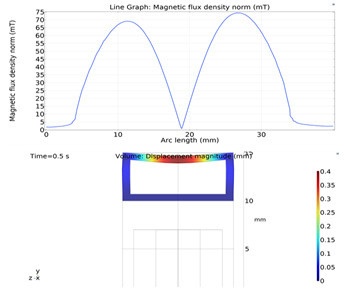	0.4
145	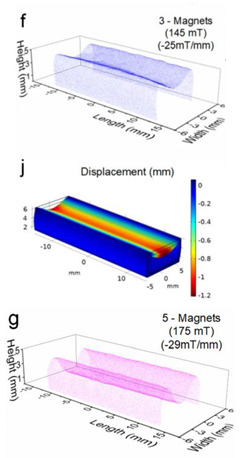	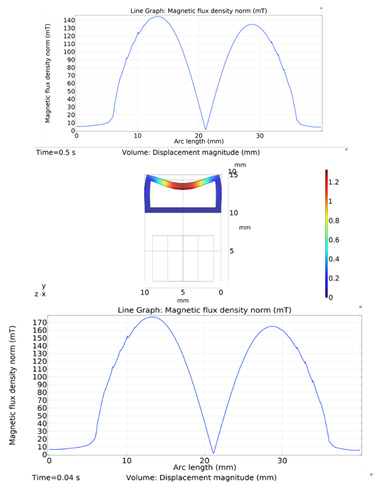	1.2
175	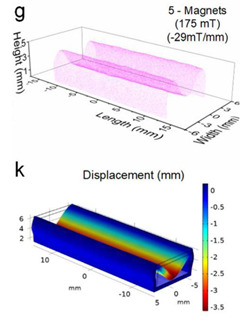	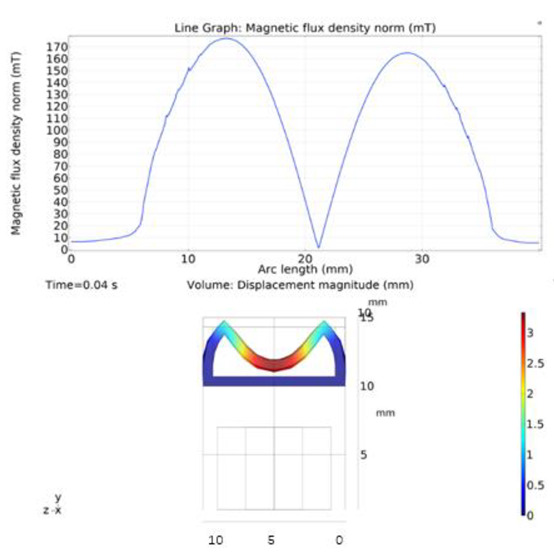	3.4

**Table 4:** Percent error of the flow rates between this study and benchmark study [31].

Magnetic Flux Density (mT)	Magnetic Flux Density (mT)	Flow Rate from Validation Case (µL/s)	Percentage of Error (%)
150	4.8	4.6	4.16
175	7.3	7.3	0
200	20.9	21.3	1.91
225	25.9	25.8	0.39

**Table 5:** Percent error of the flow rates between this study and parametric study [31].

Parameters	Design Values	Values for Parametric Study
Height of the pump chamber	0.900 mm	0.900, 0.905, 0.910, 0.915, 0.920 mm
Thickness of the upper wall	0.100 mm	0.100, 0.110, 0.120, 0.130, 0.140 mm
Elastic modulus	1.200 MPa	1.200, 5.000, 10.000, 15.000, 20.000, 25.000, 30.000, 35.000 MPa
Valve spacing distance	3.204 mm	3.204, 3.214, 3.224, 3.234, 3.244 mm
Poisson’s ratio of the valve	0.450	0.250, 0.300, 0.350, 0.400, 0.450
Length of the Valve tip	0.560 mm	0.560, 0.570, 0.580, 0.590, 0.600 mm
Poisson’s ratio of the upper wall	0.450	0.250, 0.300, 0.350, 0.400, 0.450

**Table 6:** Optimal model parameters.

Parameters	Original Model Values (mm)	Optimal Model Values (mm)
Valve spacing distance	3.204	3.244
Height of the pump chamber	0.90	0.92

**Table 7:** Performance comparison between basic model and optimal model.

Parameters	Original Design	Optimal Design	Net pumped Volume of Fluid Increased (%)
Net pumped volume (µL)	2.10	2.45	16.67

## References

[1] Chin C.D., Linder V., Sia S.K. (2012). Commercialization of microfluidic point-of-care diagnostic devices. Lab Chip.

[2] Whitesides G.M. (2006). The origins and the future of microfluidics. Nature.

[3] Thorsen T., Maerkl S.J., Quake S.R. (2002). Microfluidic Large-Scale Integration. Science.

[4] Liu J., Hansen C., Quake S.R. (2003). Solving the ‘world-to-chip’ interface problem with a microfluidic matrix. Anal. Chem..

[5] Bong K.W., Chapin S.C., Pregibon D.C., Baah D., Floyd-Smith T.M., Doyle P.S. (2011). Compressed-air flow control system. Lab Chip.

[6] Streets A.M., Huang Y. (2013). Chip in a lab: Microfluidics for next generation life science research. Biomicrofluidics.

[7] Skafte-Pedersen P., Sabourin D., Dufva M., Snakenborg D. (2009). Multi-channel peristaltic pump for microfluidic applications featuring monolithic PDMS inlay. Lab Chip.

[8] Calautit K., Nasir D.S., Hughes B.R. (2021). Low power energy harvesting systems: State of the art and future challenges. Renew. Sustain. Energy Rev..

[9] Lee M., Park T., Kim C., Park S.-M. (2020). Characterization of a magneto-active membrane actuator comprising hard magnetic particles with varying crosslinking degrees. Mater. Des..

[10] Wu C., Zhang Q., Fan X., Song Y., Zheng Q. (2019). Smart magnetorheological elastomer peristaltic pump. J. Intell. Mater. Syst. Struct..

[11] Giraldi L., Pomet J.-B. (2016). Local Controllability of the Two-Link Magneto-Elastic Micro-Swimmer. IEEE Trans. Autom. Control.

[12] Díez A.G., Tubio C.R., Etxebarria J.G., Mendez S.L. (2021). Magnetorheological Elastomer Based Materials and Devices: State of the Art and Future Perspectives. Adv. Eng. Mater..

[13] Arribas P., Sánchez P. (1989). We are IntechOpen, the world’s Leading Publisher of Open Access Books Built by Scientists, for scientists TOP 1%. Intech.

[14] An H.-N., Sun B., Picken S.J., Mendes E. (2012). Long Time Response of Soft Magnetorheological Gels. J. Phys. Chem. B.

[15] Carlson J., Jolly M.R. (2000). MR fluid, foam and elastomer devices. Mechatronics.

[16] Davis L.C. (1999). Model of magnetorheological elastomers. J. Appl. Phys..

[17] Ginder J.M., Davis L.C. (1994). Shear stresses in magnetorheological fluids: Role of magnetic saturation. Appl. Phys. Lett..

[18] Gong X., Xu Y., Xuan S., Guo C., Zong L. (2012). The investigation on the nonlinearity of plasticine-like magnetorheological material under oscillatory shear rheometry. J. Rheol..

[19] Oliveira F., Botto M.A., Morais P., Suleman A. (2017). Semi-active structural vibration control of base-isolated buildings using magnetorheological dampers. J. Low Freq. Noise Vib. Act. Control.

[20] Rabinow J. (1948). The magnetic fluid clutch. Electr. Eng..

[21] Rodriguez-Lopez J., Elvira L., Resa P., De Espinosa F.M. (2013). Sound attenuation in magnetorheological fluids. J. Phys. D Appl. Phys..

[22] Song W.-L., Li D.-H., Tao Y., Wang N., Xiu S.-C. (2017). Simulation and experimentation of a magnetorheological brake with adjustable gap. J. Intell. Mater. Syst. Struct..

[23] Sun S., Peng X., Guo Z. (2014). Study on Macroscopic and Microscopic Mechanical Behavior of Magnetorheological Elastomers by Representative Volume Element Approach. Adv. Condens. Matter Phys..

[24] Amirouche F., Zhou Y., Johnson T. (2009). Current micropump technologies and their biomedical applications. Microsyst. Technol..

[25] Laser D.J., Santiago J.G. (2004). A review of micropumps. J. Micromech. Microeng..

[26] Nguyen N.-T. (2011). Micro-magnetofluidics: Interactions between magnetism and fluid flow on the microscale. Microfluid. Nanofluid..

[27] Behrooz M., Gordaninejad F. (2016). Three-dimensional study of a one-way, flexible magnetorheological elastomer-based micro fluid transport system. Smart Mater. Struct..

[28] Behrooz M., Gordaninejad F. (2016). A flexible micro fluid transport system featuring magnetorheological elastomer. Smart Mater. Struct..

[29] Stork M., Mayer D. (2018). Peristaltic Pump with Magnetoelastic Drive. IEEE Trans. Magn..

[30] Ehsani A., Nejat A. (2017). Conceptual design and performance analysis of a novel flexible-valve micropump using magneto-fluid-solid interaction. Smart Mater. Struct..

[31] Cao X., Xuan S., Hu T., Gong X. (2020). 3D printing-assistant method for magneto-active pulse pump: Experiment, simulation, and deformation theory. Appl. Phys. Lett..

